# Exploring Experiences Utilizing Technology of Dementia Caregivers in Hospice

**DOI:** 10.1093/geroni/igaf122.2801

**Published:** 2025-12-31

**Authors:** Hannah Cho, Justine Sefcik, George Demiris

**Affiliations:** University of Pennsylvania, Philadelphia, Pennsylvania, United States; Drexel University College of Nursing and Health Professions, Philadelphia, Pennsylvania, United States; University of Pennsylvania, Philadelphia, Pennsylvania, United States

## Abstract

Technology-mediated interventions among dementia caregivers in hospice settings offer significant potential to transform end-of-life care delivery and alleviate caregiver burden. As family members navigate complex end-of-life tasks while facing unique circumstances like anticipatory grief and life reprioritization, they experience both rewards and significant stress from dementia’s unpredictable trajectory. Despite technology’s rapid reshaping of healthcare delivery, research examining how these technologies function specifically within dementia hospice care remains notably limited. This research aims to explore the experiences of dementia caregivers utilizing technology during hospice care. This qualitative descriptive study was guided by two frameworks Technology-enabled caregiving in the home and the Pearlin Stress Model. We conducted semi-structured interviews with 10 adult caregivers (mean age 63.6 ± 13.27, female 70%) who provided care for PWD during hospice care at home (n = 8) or in nursing homes (n = 2). Through a direct content analysis, results revealed three distinct patterns of technology use among dementia caregivers in hospice: administrative management, social support, and access to caregiving resources. Most of the caregivers emphasized that gaining online resources or training information was helpful in early dementia; however, later, during hospice care, they used technology more for practical care coordination tools such as reminders for medications and communication tools among families. These changes in technology utilization highlight the critical need for adaptive technology-mediated interventions that effectively support transitions in care, particularly assisting caregivers and their PWD as goals shift from aggressive measures to a comfort-based approach during hospice.

